# Supplementary figures and images for: Antibody response to Aedes aegypti D7L1 + 2 salivary proteins as marker of aggregate vector exposure and correlate of dengue virus susceptibility
Source: PLoS Negl Trop Dis. 2025 Oct 29;19(10):e0013099. doi: 10.1371/journal.pntd.0013099 (PMC12588518; doi:10.1371/journal.pntd.0013099)

S1 Fig

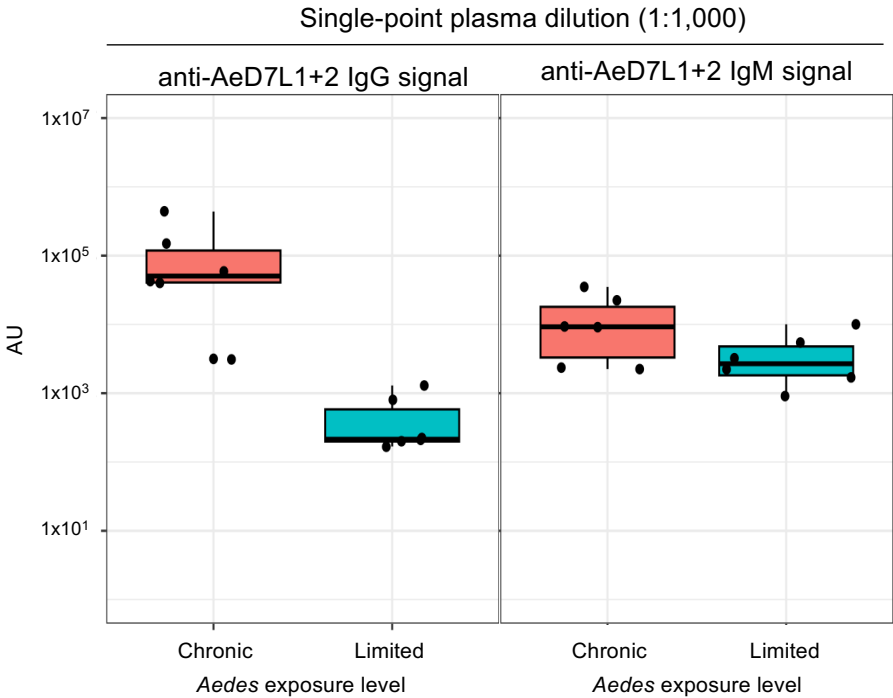

Supplement: S1 Fig — A) Anti-AeD7L1 + 2 IgG and IgM values for the pooled plasma samples from Fig 1A. B) ROC analyses of the sensitivity of these antibody levels in delineating exposure using pooled samples. (PDF) [file pntd.0013099.s001.pdf]

S2 Fig

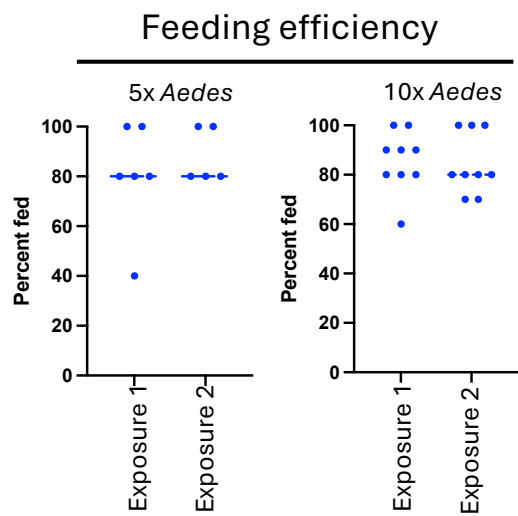

Supplement: S2 Fig — Mosquito engorgement data for first and second Aedes aegypti exposures. Percentage of mosquitoes out of 5 or 10 that fed during each exposure. (PDF) [file pntd.0013099.s002.pdf]

S3 Fig

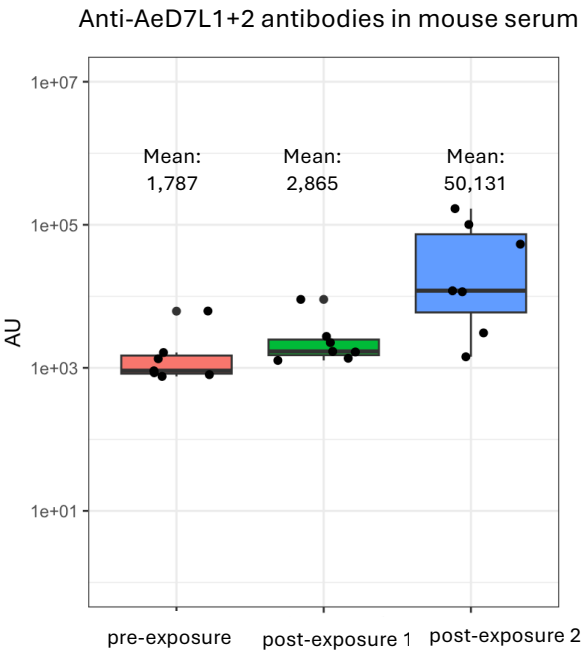

Supplement: S3 Fig — 7 female BALB/C mice were fed on by approximately 100 Aedes aegypti mosquitoes twice, once on day 0 and once on day 41. Serum was collected before exposure and 10 days after each exposure. Values analyzed using Friedman’s test and a Dunn’s multiple comparisons test. Antibody levels increased significantly after the second Aedes exposure (p = 0.0066). (PDF) [file pntd.0013099.s003.pdf]

S4 Fig

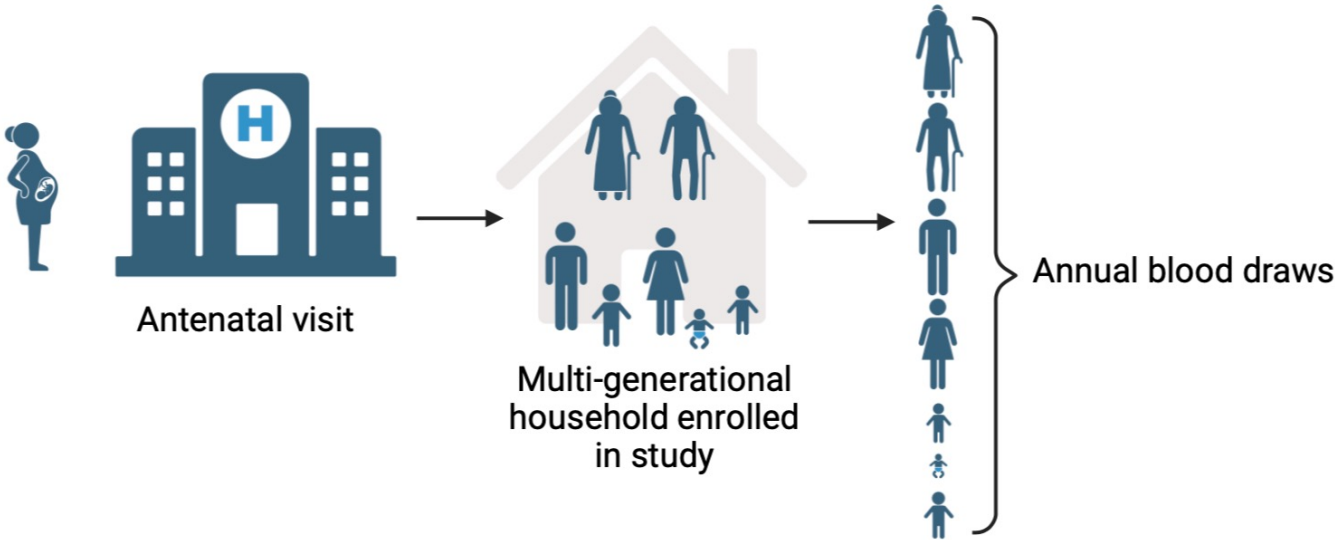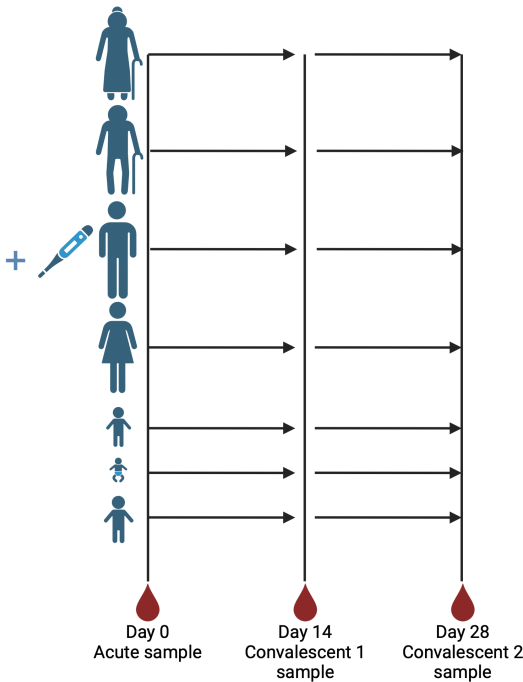

Supplement: S4 Fig — Schematic of KFCS enrollment and sample collection. Figure Created in BioRender. Bahr, L. (2025) https://BioRender.com/2o64swx. Multigenerational households are enrolled starting with a mother from the household in their third trimester of pregnancy. Cord blood samples are taken from the newborn at birth and enrollment samples are taken from the household members. Longitudinal samples are taken annually. (PDF) [file pntd.0013099.s004.pdf]

S5 Fig

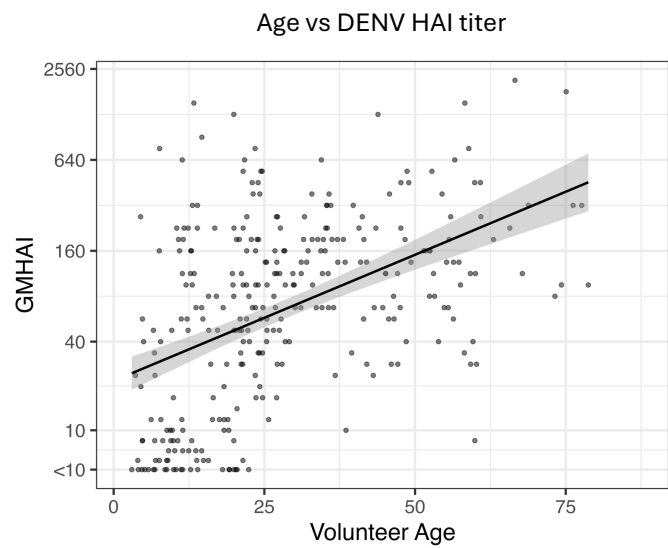

Supplement: S5 Fig — Annual anti-DENV HAI titers compared to subject age at time of sample. Regression analysis represented by the solid black line, gray shaded area represents 95% confidence interval. Regression analysis suggests that there is a 0.05 (0.04-0.06) log2 unit increase in GMHAI titers for every year of age increase (p < .001). (PDF) [file pntd.0013099.s005.pdf]
