# Supplementary material for: Antibody response to Aedes aegypti D7L1 + 2 salivary proteins as marker of aggregate vector exposure and correlate of dengue virus susceptibility
Source: PLoS Negl Trop Dis. 2025 Oct 29;19(10):e0013099. doi: 10.1371/journal.pntd.0013099 (PMC12588518; doi:10.1371/journal.pntd.0013099)
Supplement: Attachment — Submitted filename: response_to_reviewers_03SEP25.pdf [file pntd.0013099.s008.pdf]

Response to reviewers

Reviewer #1:

Is it possible that the sequence of D7 present in the laboratory-reared mosquitoes was not equivalent to the recombinant D7 and the D7 present in endemic Thailand? That would explain why the assay failed to detect anti-D7 antibodies in the New York population that was experimentally challenged with mosquito bites. Could these samples be analyzed for a broader target such as the less than ideal MSP? It's hard to believe that there would be no seroconversion to any mosquito saliva target.

We wish to thank the reviewer for this intriguing question. To confirm that our lab-reared *Aedes* mosquitos are capable of eliciting a AeD7L1+2 -specific immune response in mammalian hosts after feeding, we performed an additional/new animal study wherein mice were fed-upon by ~100 *Aedes aegypti* mosquitos twice over the course of 60 days. Serum was collected from the *Aedes* exposed animals before both feedings and 10 days after the final feeding. As now shown in the new Supplemental Figure 3, this exposure scheme resulted in the generation of robust anti-AeD7L1+2 IgG antibody titers in the animals, but only after the second exposure. Taking into account the physical size of the animals and the number of mosquitos involved in the exposure, this assay represents a significantly higher antigen exposure (per unit weight or volume) than what our human volunteers experienced in their challenge study. Accordingly, we posit that the exposure intensity experienced by our human volunteers was insufficient to generate anti-AeD7L1+2 immunity.

We also wish to thank the reviewer for their interesting suggestion to broaden the antigen panel utilized in this study to include other MSP. However, the focus of this study is specifically anti-AeD7L1+2 immunity, not anti-MSP immunity in general. As such, we feel that this additional analysis falls outside the scope of our current study.

Lines 268-279: More work/explanation is needed to confirm that the New York cohort didn't seroconvert. Are the D7 sequences the same in the lab-reared mosquito as the recombinant protein? Mosquito saliva proteins evolve quickly and a single amino acid mismatch could have undermined the assay in this experiment. If the sequences are different, it would be helpful to run a separate assay either with updated D7 or a broader target like MSP to confirm that they didn't seroconvert.

Please see above response.

Was hypersensitivity to experimental mosquito bites measured in the New York population? Those who were hypersensitive have seroconverted to mosquito saliva proteins in the past and should seroconvert again, although it's possible that the Ae. aegypti D7 may activate B cells that recognize a similar but not identical D7 from North American mosquitoes – a type of original antigenic sin.

No overt hypersensitivity - as defined as an exaggerated response *Aedes* exposure - was observed in subjects enrolled in the mosquito human challenge study. Selection criteria for this study excluded individuals with severe hypersensitivity to insect bites as well as individuals with recent travel to an *Aedes* endemic area. We have added this information to our revised manuscript (lines 400 – 419)

Lines 244-245 and 322-332: Does the data confirm that there is a relationship between DENV infection and anti-D7 antibodies or is it possible that the assay is an indirect proxy for another anti-saliva response that influences DENV infection? How does DENV influence immunological memory? SARS-CoV-2, measles,

etc. can negatively influence memory B cells. Is it possible that repeated DENV infection undermines the anti-mosquito saliva B cell population?

We thank the reviewer for this intriguing question. We don't feel comfortable at this point making any mechanistic claims regarding the relationship between anti-MSP/anti-AeD7L1+2 IgG titers and an individual's susceptibility to DENV infection. The data presented in this manuscript are descriptive in nature and strongly suggest that subsequent mechanistic studies should be undertaken to explore potential mechanistic relationships. Should such a relationship exist – which we (and others) are attempting to dissect in additional studies - we agree that anti-AeD7L12 IgG titers might be a proxy for antibodies directed against other MSP antigens that might be directly responsible for modulating DENV infectivity. We have re-emphasized these limitations the discussion section of our revised submission (lines 370-382)

The 2009 WHO classification system no longer includes DHF/DSS and instead focused on severe dengue.

We wish to thank the reviewer for this observation. DHF/DSS definitions are still broadly used in Thailand where the cohort study described in this study is conducted, but we have revised our manuscript to remove the DHF/DSS distinction (line 86).

Is development of an IgG response linear or will overexposure of an antigen lead to a decrease in antibody production?

We thank the reviewer for this intriguing question. While there is evidence that persistent/chronic antigen exposure can lead to immune anergy or tolerance we do not have the data to demonstrate this exact mechanism is at-play in our cohort and analysis. The new animal exposure data now shown in Supplemental Figure 2 suggests that significant exposure is required to elicit anti-AeD7L1+2 IgG antibodies, but we have not yet explored this phenomenon further to see if titers drop with additional feedings. However, we do wish to note that DENV HAI titers in this same group of volunteers do not demonstrate a decrease with age, as shown in the new Supplemental Figure 5. This is an imperfect comparison, however, as we would predict that the kinetics and trajectory of anti-DENV immunity would be very different than anti-AeD7L1+2 immunity due to the episodic and discrete nature of DENV exposure/infection. We posit that this would be different than the continual/chronic nature of AeD7L1+2 exposure in individuals living in *Aedes* endemic regions of the world.

On line 162 it suggests that the study continued through day 84 but the figure lists that it ends on day 77. Please clarify in the text or in the figure.

We thank the reviewer for this feedback and have clarified in the text the length of the study (lines 171 to 176).

Lines 201-204: What season is Ae. aegypti most abundant in this region? Please describe.

***Aedes aegypti* are most abundant during the rainy seasons in Thailand (roughly May to October). We appreciate the reviewer for highlighting this omission in our initial submission and have further clarified this in our revised text (lines 229-231).**

Reviewer #2:

The assay details on the serological assay are missing (what sequences) - important since other groups reported the suitability of the D7 protein to determine recent exposures. Since the assay applied here does not have the resolution to determine recent exposures, the conclusions on susceptibility to DENV is not secured.

We thank the reviewer for pointing this out. We have added the GenBank ID for the AeD7L1 and AeD7L2 proteins to the methods section (lines 442-443).

The data do not support the conclusion and title of the manuscript (see detailed comments). The figure captions are missing making it harder to review the results. Key data such as changes in HAI titer as function of age are not presented. The data are log10 or log 2 transformed but the scales are always linear.

Please see comments below. In addition, we apologize for the inconsistent axis labeling, which have been updated in our revised submission. We also wish to highlight that the addition of the new Supplemental Figure 5, which presents the requested DENV HAI titers vs age in the volunteers/samples included in this study.

Figure 2: The data demonstrate that the D7L1+2 "probes" used in this study (sequences were not provided) are not suited for detecting recent exposures. This is surprising since one of the co-authors contributed to a study demonstrating the suitability of D7L plate antigens in an ELISA-based assay (doi: 10.3389/fimmu.2024.1368066. PMID: 38751433). Is the discrepancy of the results due to the high sensitivity of the MSD platform (suboptimal signal-to-noise ratio)?

We wish to thank the reviewer for this astute observation. *Chea et al*. indeed demonstrated that there was a significant change in anti-AeD7L1+2 antibody titers in sequential samples collected from volunteers living in Cambodia across the wet and dry season. This was an observation that we were able to recapitulate on our analysis (Figure 3C), albeit with less statistical power due to the lack of paired samples from the same individuals across multiple seasons. Sample collection in the cohort presented in this study occurs only yearly or upon enrollment or illness investigation. We were able to generate the data in Figure 3C due to fortuitous asynchronous enrollment of some individuals in our study. In their manuscript, *Chea et al*. make no concrete claims regarding the timing and/or magnitude of exposure required to elicit changes in anti-AeD7L1+2 antibody titers. Accordingly, we posit that the observations presented in our manuscript are concordant with those presented by *Chea et al*.

Figure 3: The correlation between age and antibody titers suffers from low sample size for the higher ages (n=7 based on Panel A). The authors discuss later that the lower antibody titers in the older participants may be due to immunosenescence. It would have been important to show a parallel figure with the HAI titers. Besides immunosenescence, there are other explanations such as induction of tolerance or, more likely, older individuals having lower activity levels outside the house and therefore lower chance for exposure.

We thank the reviewers for their input on alternative explanations for the lower antibody titers observed in older individuals and we have added the new Supplemental Figure 5 to our revised submission to directly address this consideration. Unlike what was observed for the anti-AeD7L1+2 IgG titers in our cohort, DENV HAI titers overwhelmingly increased with age in the

volunteers/samples included in this analysis. We have also expanded upon the suggested alternative explanations highlighted by the reviewer in the discussion section of our revised submission (lines 318 – 320).

Figure 4: The causality of seroconversion to DENV (no details provided whether this is solely based on HAI titers or whether other serology was established such as anti-NS1 tests) is not secured. It seems reasonable that DENV infected individuals have lower activity resulting in less exposure to mosquito bites. Since the antigenic probes using in this MSD-assay does not have sufficient resolution to determine recent exposure, the data cannot support the conclusions drawn by the authors.

We would like to emphasize that we are making no claims as to mechanism or causality in this manuscript. This is an observational study, and addressing these considerations would require a different study design and analysis strategy. We would also like to reiterate that these were seroconversions that were identified using HAI assays alone, not infection events. We have re-emphases these consideration and limitations in our revised submission.

The conclusions are not supported by the data. causality is not demonstrated and the sample sizes for some of the age groups is low.

As stated above we are not seeking to establish or demonstrate a causal relationship between the immune parameters assessed in this study or any specific clinical outcome. Sample sizes were based on samples available from the study that fit the parameters needed for this study.

The supplementary data do not add additional information to the manuscript. Other details such as in the Materials and Methods and in the result section (e.g. HAI titers) would be important to add.

We appreciate the reviewer pointing this out and have added more detail to the Methods section about HAI.

Reviewer #3:

Justify the choice of specific time points Days 35 and 77 in the human challenge, are these based on expected immune response?

Days 35 and 77 were chosen based on anticipated immune response following mosquito exposure, we have clarified this consideration in the text of our revised submission.

Briefly state how mosquitoes were screened to identify pathogen free status in the lab setting?

Mosquitoes were screened via PCR for a select panel of arboviral pathogens DENV, Chikungunya virus, Zika virus, Yellow fever virus, Mayaro virus, West Nile virus, and Usutu virus. We have clarified this in the methods section of our revised submission (line 408 – 409).

Exposure to only 5–10 mosquitoes might be insufficient, so justify in which basis the number was selected ?

We agree with the reviewer that exposure to 5-10 mosquitos was likely insufficient to induce *de novo* humoral immunity to AeD7L1+2. This supposition is supported by the new mouse exposure data now shown in Supplemental Figure 3, where animals seroconverted to AeD7L1+2 only after two sequential exposures to ~100 mosquitos. However, the decision to utilize 5-10 mosquitos for this human

exposure study was made prior to the generation of these animal data and was motivated by a desire to assess the ability of the assay to determine proximal exposure to a small number of *Aedes* mosquitos. In addition, this decision was made in collaboration with the SUNY Upstate Institutional Review Board in order to minimize volunteer discomfort.

The statement about anti D7L1+2 antibodies may be protective is too speculative for observational data. Clarify that anti D7L1+2 IgG serves better as a population level surveillance tool not an individual risk marker.

We agree that anti-AeD7L1+2 antibodies serve best as an aggregate population level marker of exposure and have clarified the text to best reflect this fact.

Include interpretation that lower antibody levels could reflect general immune immaturity or may be waning immunity.

We have re-emphasized this very reasonable alternative explanation in the discussion portion of our revised manuscript.

Consider co-infections or household transmission chains as additional factors.

We wish to thank the reviewer for this intriguing suggestion. However, as we utilized seroconversions and not acute infection events to identify DENV infections in our cohort, tracking factors such as household transmission and co-infections difficult to infer.
