# Supplementary material for: Antibody response to Aedes aegypti D7L1 + 2 salivary proteins as marker of aggregate vector exposure and correlate of dengue virus susceptibility
Source: PLoS Negl Trop Dis. 2025 Oct 29;19(10):e0013099. doi: 10.1371/journal.pntd.0013099 (PMC12588518; doi:10.1371/journal.pntd.0013099)
Supplement: Attachment — Submitted filename: Review PNTD-D-25-00674.docx [file pntd.0013099.s007.docx]

The authors developed a serologic assay to detect IgG antibodies against Ae. aegypti D7 saliva proteins. This was done to better understand individual exposure and determine if this risk correlates with arboviral infection. The assay was unable to reveal when individuals were exposed to recent, low levels of mosquito bites, but was able to detect antibodies in individuals from endemic regions. Interestingly, the signal ebbed and flowed based on the mosquito biting season. There was also an inverse correlation between anti-D7 antibodies and infection burden in households.

Major

-Is it possible that the sequence of D7 present in the laboratory-reared mosquitoes was not equivalent to the recombinant D7 and the D7 present in endemic Thailand? That would explain why the assay failed to detect anti-D7 antibodies in the New York population that was experimentally challenged with mosquito bites. Could these samples be analyzed for a broader target such as the less than ideal MSP? It’s hard to believe that there would be no seroconversion to any mosquito saliva target.

-Lines 268-279: More work/explanation is needed to confirm that the New York cohort didn’t seroconvert. Are the D7 sequences the same in the lab-reared mosquito as the recombinant protein? Mosquito saliva proteins evolve quickly and a single amino acid mismatch could have undermined the assay in this experiment. If the sequences are different, it would be helpful to run a separate assay either with updated D7 or a broader target like MSP to confirm that they didn’t seroconvert.

-Was hypersensitivity to experimental mosquito bites measured in the New York population? Those who were hypersensitive have seroconverted to mosquito saliva proteins in the past and should seroconvert again, although it’s possible that the Ae. aegypti D7 may activate B cells that recognize a similar but not identical D7 from North American mosquitoes – a type of original antigenic sin.

-Lines 244-245 and 322-332: Does the data confirm that there is a relationship between DENV infection and anti-D7 antibodies or is it possible that the assay is an indirect proxy for another anti-saliva response that influences DENV infection? How does DENV influence immunological memory? SARS-CoV-2, measles, etc. can negatively influence memory B cells. Is it possible that repeated DENV infection undermines the anti-mosquito saliva B cell population?

Minor

-The 2009 WHO classification system no longer includes DHF/DSS and instead focused on severe dengue.

-Is development of an IgG response linear or will overexposure of an antigen lead to a decrease in antibody production?

-On line 162 it suggests that the study continued through day 84 but the figure lists that it ends on day 77. Please clarify in the text or in the figure.

-Lines 201-204: What season is Ae. aegypti most abundant in this region? Please describe.
